# Supplementary material for: Long-term trends in the intensity and relative toxicity of herbicide use
Source: Nat Commun. 2017 Apr 10;8:14865. doi: 10.1038/ncomms14865 (PMC5394230; doi:10.1038/ncomms14865)
Supplement: Supplementary Information — Supplementary Figures [file ncomms14865-s1.pdf]

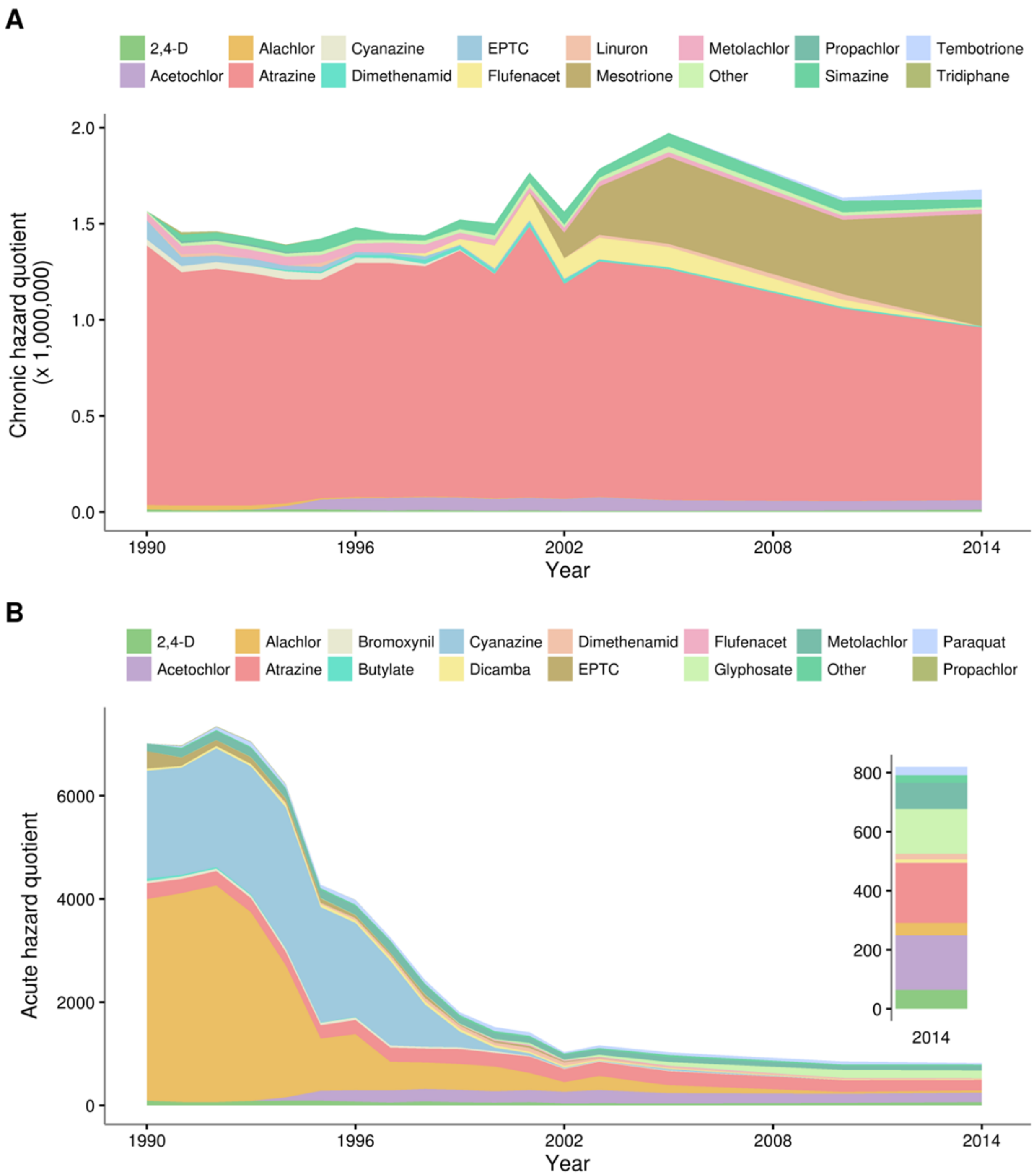

Supplementary Figure 1. Chronic and acute toxicity from maize herbicide use in the United States, 1990 to 2014. (A) Chronic (24 month rat) toxicity of herbicides applied, NOEL/acre. (B) Acute mammalian toxicity of herbicides applied, LD_{50}/acre. Inset bar in panel B shows enlarged data for 2014 only, y-axis is acute hazard quotient.

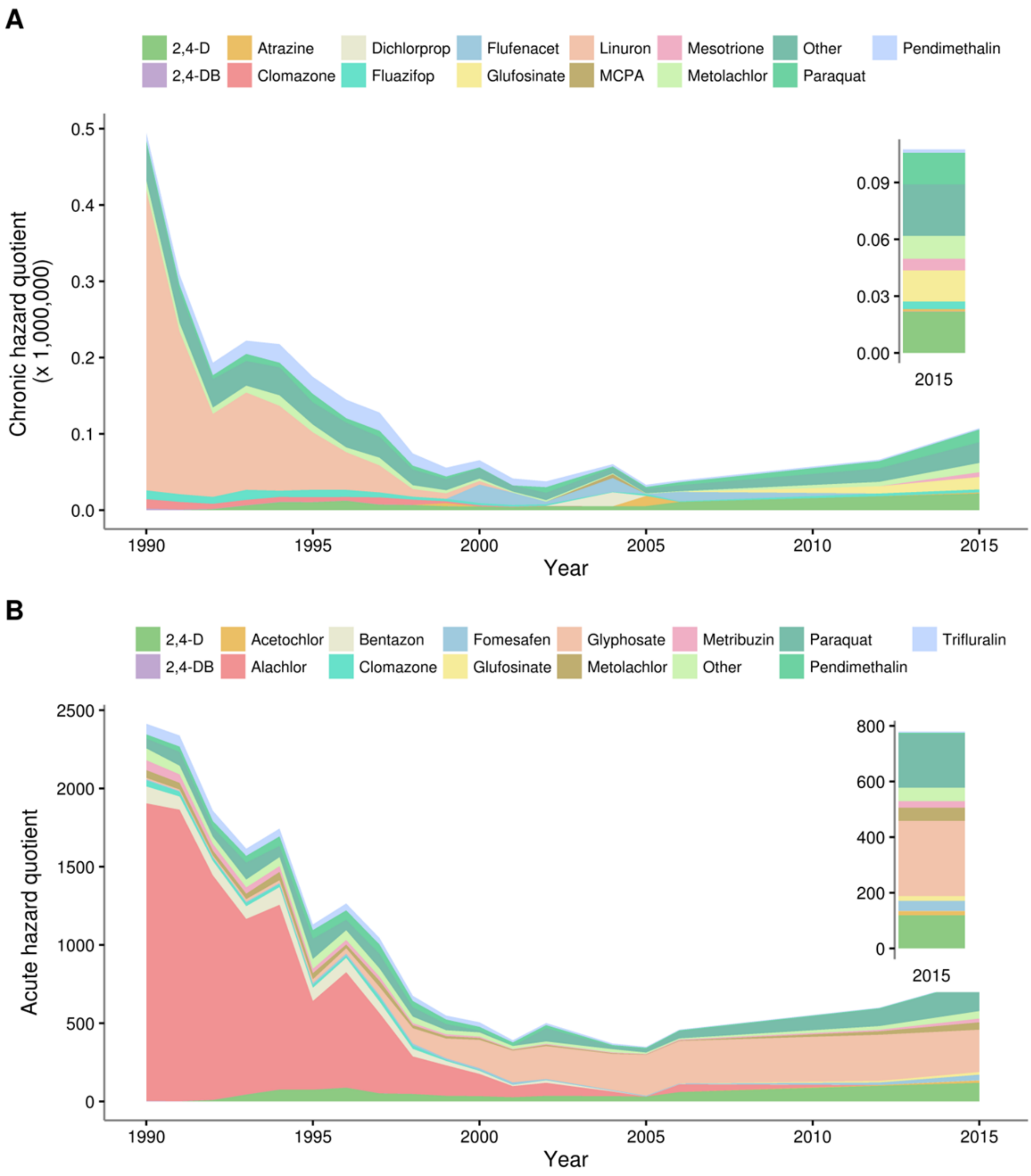

Supplementary Figure 2. Chronic and acute toxicity from soybean herbicide use in the United States, 1990 to 2014. (A) Chronic (24 month rat) toxicity of herbicides applied, NOEL/acre. (B) Acute mammalian toxicity of herbicides applied, LD_{50}/acre. Inset bars show enlarged data for 2015 only, y-axis is chronic (A) or acute (B) hazard quotient.

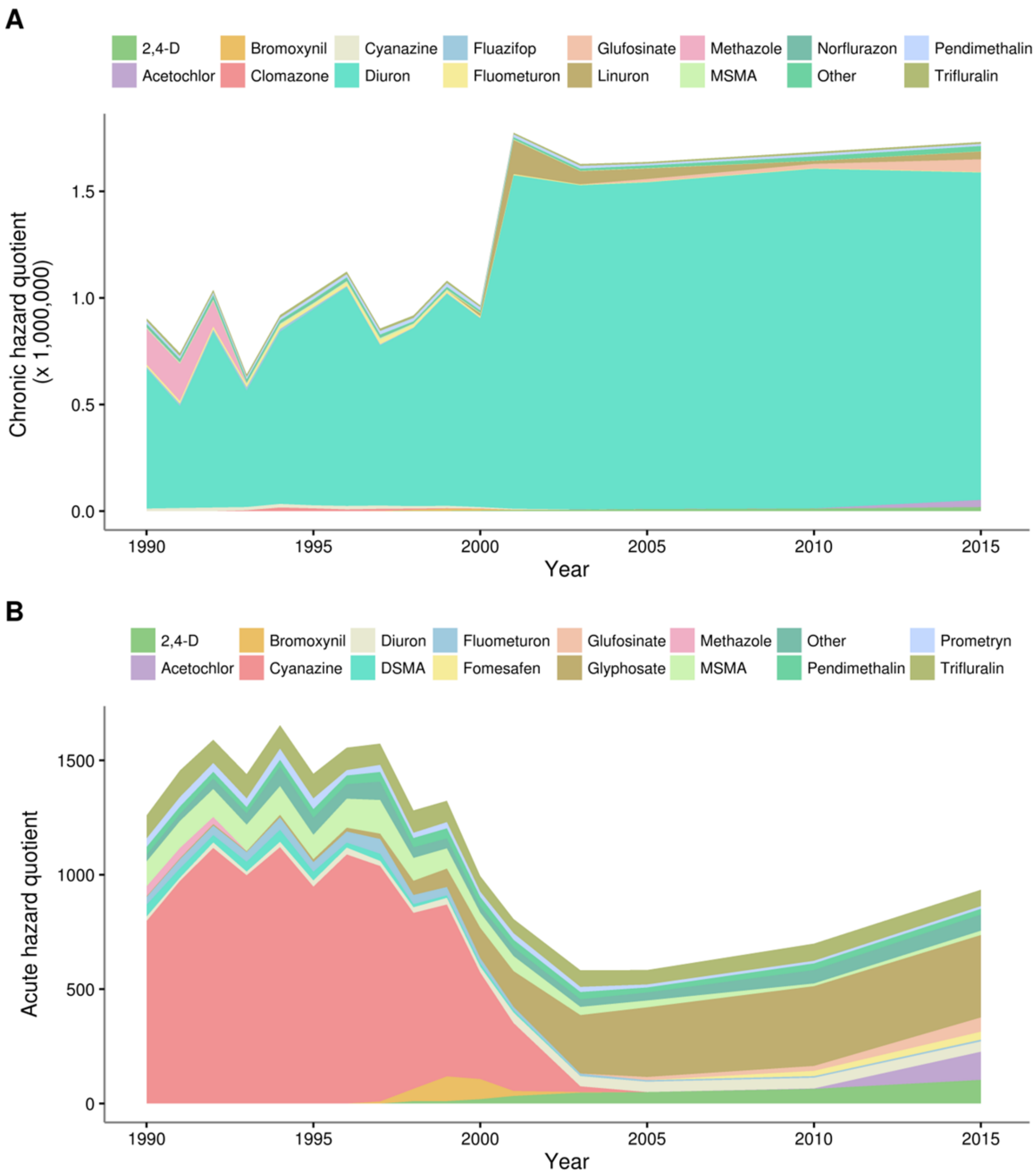

Supplementary Figure 3. Chronic and acute toxicity from cotton herbicide use in the United States, 1990 to 2015. (A) Chronic (24 month rat) toxicity of herbicides applied, NOEL/acre. (B) Acute mammalian toxicity of herbicides applied, LD_{50}/acre.

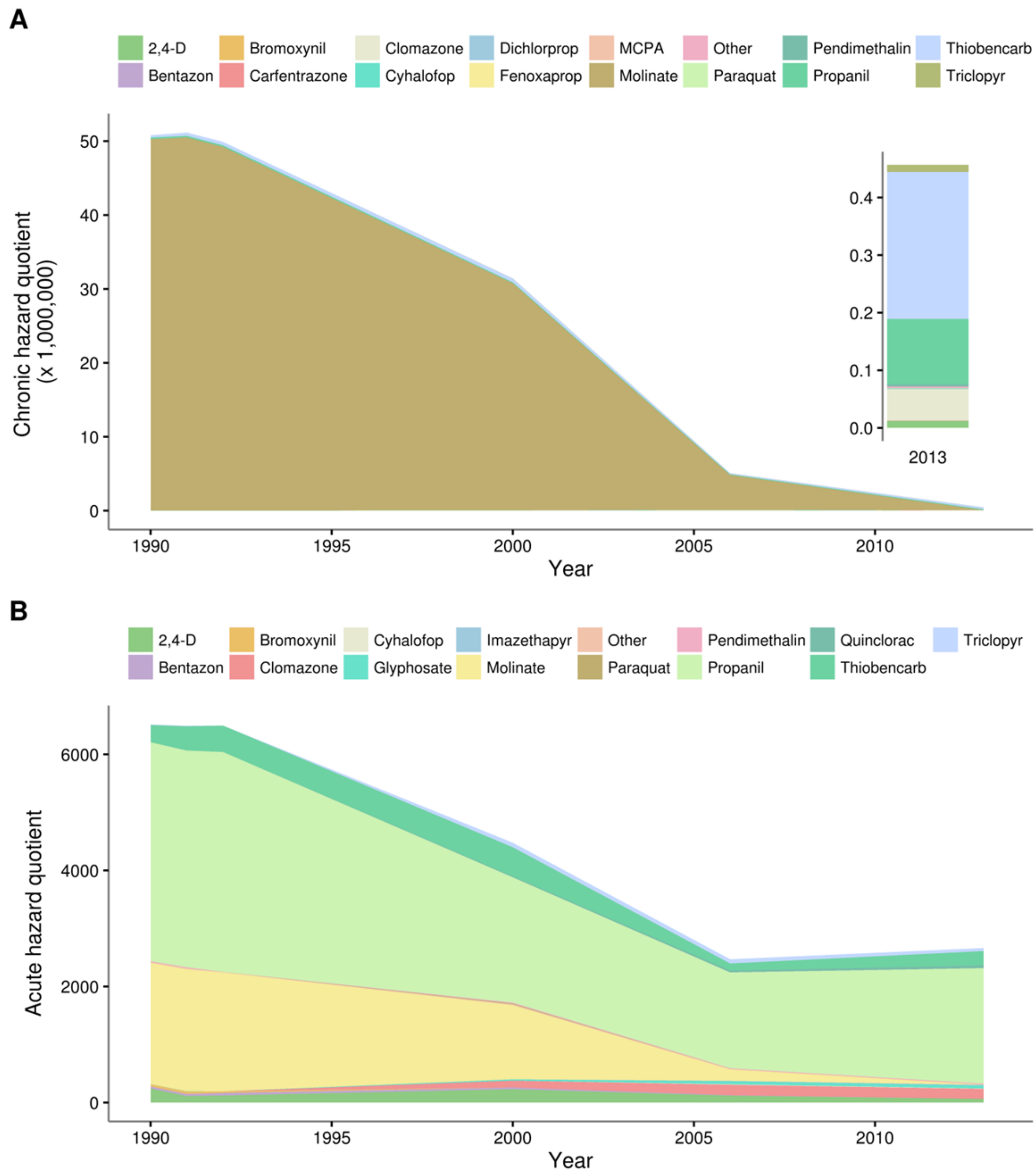

Supplementary Figure 4. Chronic and acute toxicity from rice herbicide use in the United States, 1990 to 2014. (A) Chronic (24 month rat) toxicity of herbicides applied, NOEL/acre. (B) Acute mammalian toxicity of herbicides applied, LD_{50}/acre. Inset bar in panel A shows enlarged data for 2013 only, y-axis is chronic hazard quotient.

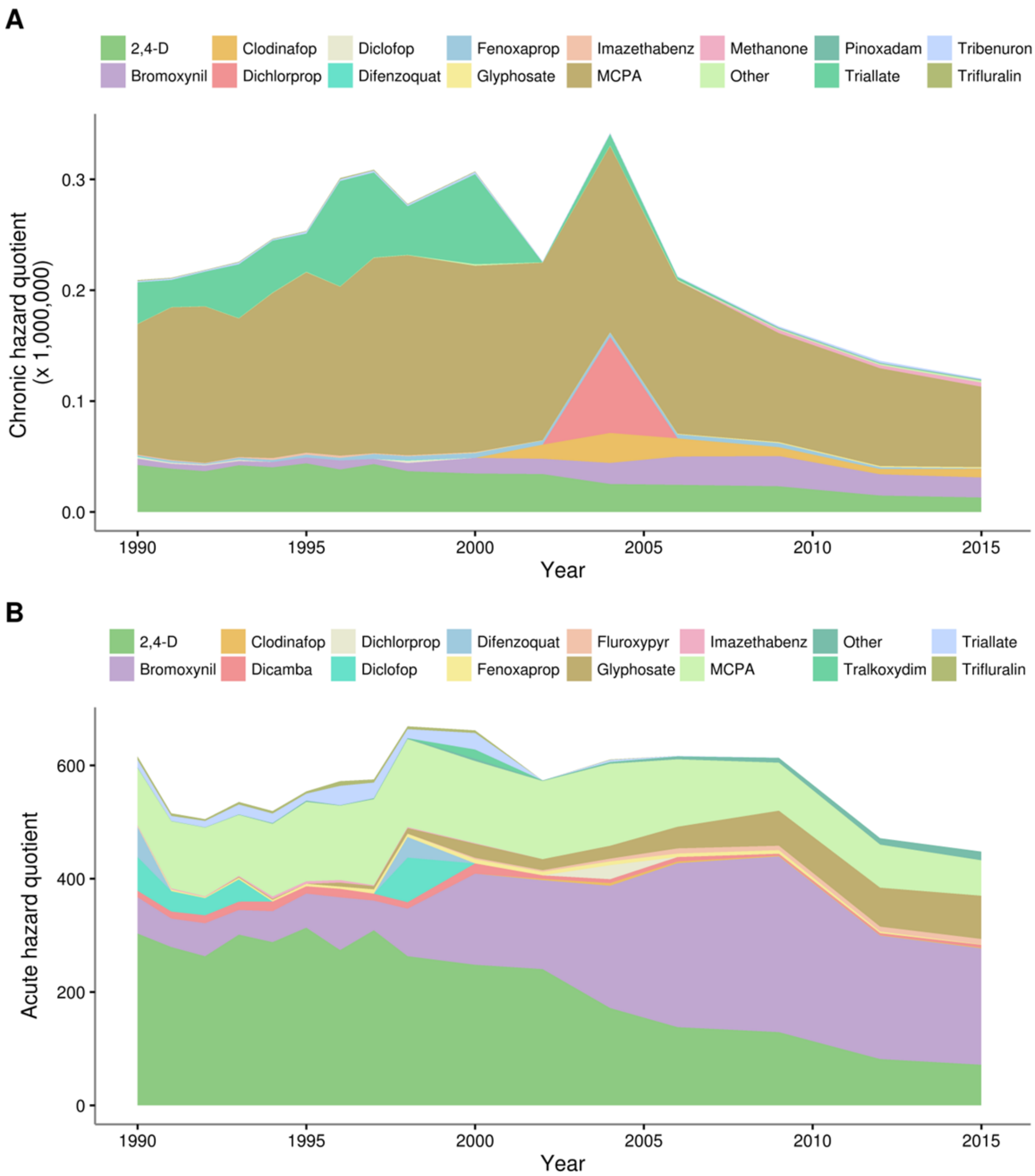

Supplementary Figure 5. Chronic and acute toxicity from spring wheat herbicide use in the United States, 1990 to 2014. (A) Chronic (24 month rat) toxicity of herbicides applied, NOEL/acre. (B) Acute mammalian toxicity of herbicides applied, LD_{50}/acre.

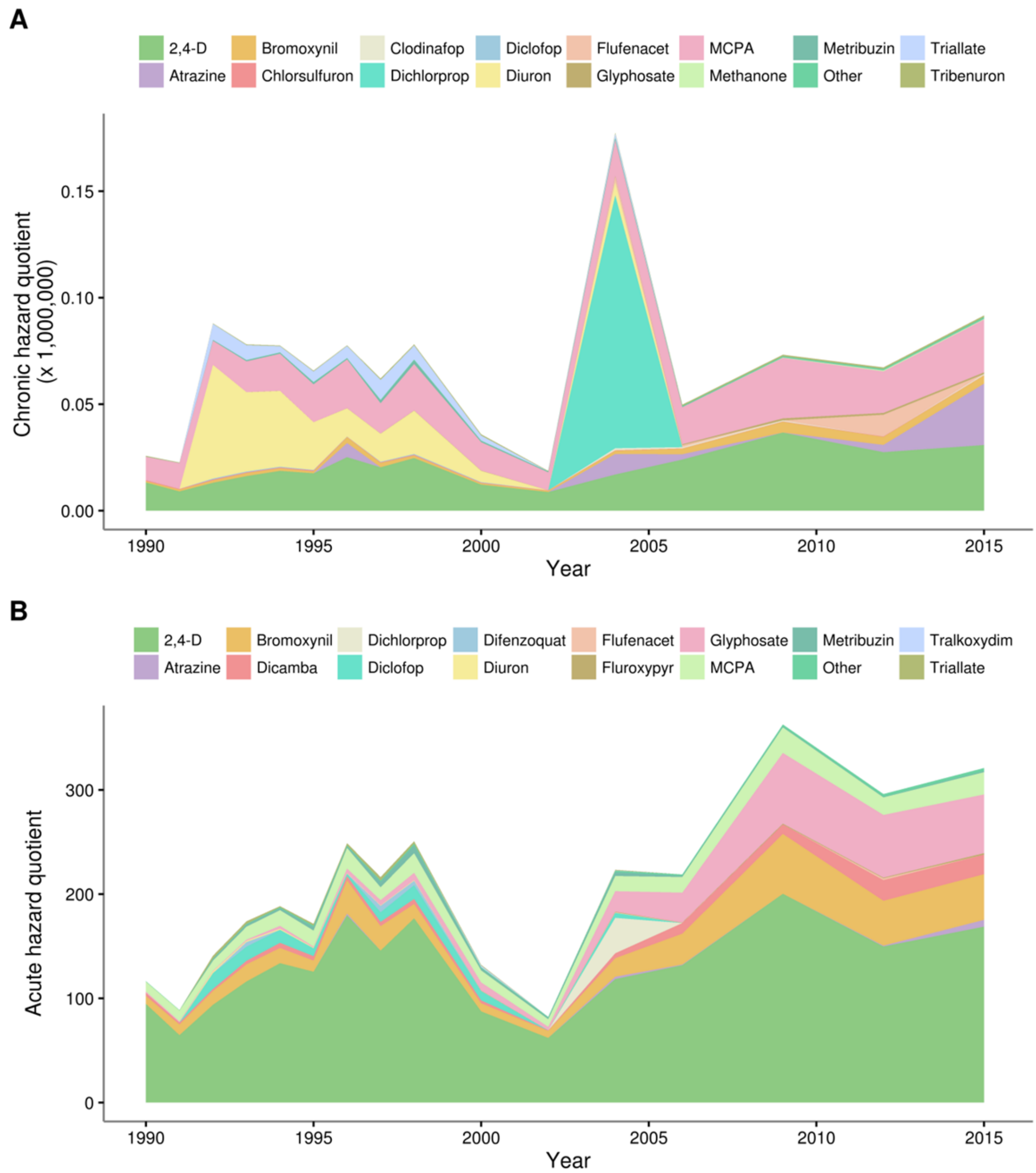

Supplementary Figure 6. Chronic and acute toxicity from winter wheat herbicide use in the United States, 1990 to 2014. (A) Chronic (24 month rat) toxicity of herbicides applied, NOEL/acre. (B) Acute mammalian toxicity of herbicides applied, LD_{50}/acre.
